# Synthesis, Characterization, Luminescent and Nonlinear Optical Responses of Nanosized ZnO

**DOI:** 10.1186/s11671-017-1934-y

**Published:** 2017-03-03

**Authors:** Volodymyr V. Multian, Andrii V. Uklein, Alexander N. Zaderko, Vadim O. Kozhanov, Olga Yu Boldyrieva, Rostyslav P. Linnik, Vladyslav V. Lisnyak, Volodymyr Ya Gayvoronsky

**Affiliations:** 1 Institute of Physics, National Academy of Science of Ukraine, 46, Prospect Nauky, Kyiv, 03680 Ukraine; 2 Faculty of Chemistry, Taras Shevchenko National University of Kyiv, 62a, Volodymyrska Str., Kyiv, 01601 Ukraine

**Keywords:** ZnO nanoparticles, Hydrolysis, NLO response, Remote optical diagnostics, Second harmonic generation

## Abstract

In this study, we report soft and solvothermal methods for synthesis of zinc oxide nanoparticles (ZnO NPs). Both methods involve a precursor and are carried out at the middle low-temperature regime. The effect of different solvents on the ZnO NPs properties was studied. The nonlinear optical (NLO) response of the NPs was analyzed by the self-action of picosecond laser pulses at 1064 nm and by second harmonic generation (SHG) of a femtosecond laser pulses pump at 800 nm. The luminescence was studied within UV-visible ranges. It was shown that the NLO response efficiency significantly depends on the solvent. The obtained SHG efficiency of small (~2 nm) ZnO NPs is comparable to the one obtained for large (~150 nm) commercial ZnO NPs. The observed results are important for the application of the ZnO NPs in biolabeling.

**Electronic supplementary material:**

The online version of this article (doi:10.1186/s11671-017-1934-y) contains supplementary material, which is available to authorized users.

## Background

Zinc oxide (ZnO) is one of the widely used materials. It is a low-cost semiconductor with a large bandgap (3.37 eV), high Wannier-Mott exciton binding energy (~60 meV), and promising luminescent properties [1–8]. The ZnO nanoparticles (NPs) and quantum dots (QDs) have found their application in the fields of sensing, LEDs, etc. They can be assembled with transparent conductive oxide waveguides for the realization all ZnO-based optoelectronic devices [9]. However, there are a few reports on synthesizing of ZnO NPs or QDs with ultra-small size; these results are reviewed in [2]. For this instance, the control synthesis of ZnO NPs is of interest because they possess electro-optical properties that differ from their bulk counterparts, being sensitive to both the NP’s size and shape.

Various methods [10–13], including synchrotron radiation-assisted spectroscopes [12, 13], have been reported for the characterization and theoretical modeling of wide bandgap oxide materials comprising defective and nanostructured ZnO [10, 11]. Chemical approaches for ZnO NPs preparation are the gas-phase techniques, sol-gel methods, evaporative decomposition of solutions, and wet chemical syntheses [1, 5]. In a mainstream of a large-scale synthesis, soft chemical methods can be chosen due to a low growth temperature and a good scale-up potential [2, 3].

To obtain small ZnO NPs, we have used a chemical method under a wet chemistry paradigm, which, by definition, involves stabilization of ZnO core with an organic amine [6, 14]. In this case, the used templating agent acted as a weak base promoting the formation of ZnO NPs [6]. The NPs’ growth condition must be carefully chosen to minimize hydrolysis at the earliest stage, which determines the ZnO core size. The ZnO nucleation and NP growth should pass under the action of the equimolar amount of hydrolytic agent (H_2_O). This condition is satisfied in the case of zinc acetate dihydrate Zn(CH_3_COO)_2_ × 2H_2_O — the substrate capable of autohydrolysis. A cascade growth of ZnO NPs can be typically prevented by keeping the hydrolysis within the absolutized solvent medium. Taking into account the fact that higher substrate concentration causes smaller ZnO core size [15, 16], one can limit the growth rate basing on the cellular effect. Besides, a high concentration of acetate complex of zinc with an organic polyfunctional amine increases the viscosity of the solution and reduces the effective size of the cell. Therefore, sufficiently high concentrations of triethanolamine, which act as a stabilizing agent for the small ZnO NPs by adsorption on the surface, could limit the growth of the large oxide NPs.

The abovementioned arguments provide background for a proper choice of the seed mixtures for the NPs growth process. The properties of the resulted NPs were compared with the reference NPs obtained by solvothermal synthesis and the large commercial NPs (90–200 nm).

In the recent studies on harmonic nanoparticles [17], the ZnO NPs have shown a high potential for the application. Such NPs with an efficient nonlinear *χ* ^(2)^ response can be imaged via the second harmonic generation (SHG) effect as the contrast mechanism. The application in biolabeling requires the NPs size reduction (at least less than 100 nm) while maintaining the same level of the SHG efficiency.

In the present work, we provide the nonlinear optical (NLO) properties and SHG efficiency study of small ZnO NPs. Their density of defects and sizes were characterized with a range of spectroscopic techniques.

## Methods

### Reagents and Materials

Triethanolamine (TEA) was purchased from Merck. Sigma-Aldrich solvents isopropanol (*i*-PrOH), ethanol (EtOH), acetonitrile (MeCN), and propylene glycol (PPG) were dried with molecular sieves and distilled. Aldrich zinc acetate dihydrate (Zn(CH_3_COO)_2_ × 2H_2_O, ≥99%) was used as received. Commercial ZnO NPs (Nanostructured & Amorphous Materials Inc., Houston, USA) were purchased for comparison with the synthesized particles. They were specified to contain ZnO NPs of 90–200 nm in size.

### Synthesis

#### Solvothermal Synthesis

The reference ZnO NPs were obtained using the thermal hydrolysis of Zn(CH_3_COO)_2_ × 2H_2_O in 1-hexadecanol (HD, ReagentPlus®, 99%, Sigma-Aldrich) smelt according to the protocol from [18]. The thermal decomposition of ZnO precursors gives ZnO NPs with an average size of 50 nm.

#### Hydrolytic Synthesis

The ZnO NPs were produced as follows: 5.56 g of TEA was dissolved in 100 ml of abs. solvent, viz. *i*-PrOH, EtOH, MeCN, and PPG. The resulting solution was added to 13.65 g of Zn(CH_3_COO)_2_ × 2H_2_O in Pyrex® glass autoclave. With constant stirring, the solution was heated to 60 °C for 2 h. At this time, there was a gradual coloring of the prepared solution to light or pale yellow during synthesis; see Additional file 1: Figure S1. A significant precipitation was observed upon cooling of the resulting solutions, excluding PPG. The dropped precipitates are colored pale yellow too. It was found that the hydrolysis at 60 °C gives the best coloration to the ZnO NPs solution. The growth of ZnO is accompanied by the luminescence inherent in NPs [19], under UV light at 365 nm (Additional file 1: Figure S2). Since the solution shows no Tyndall cone, it contains no ZnO colloidal suspensions.

### Characterization

The characterization of optical and nanostructural properties of prepared ZnO NPs was performed as follows. Ultraviolet-diffuse reflection (UV-DR) spectra of solid precipitates containing ZnO NPs and ultraviolet-visible (UV-Vis) adsorption spectra of ZnO NPs solutions were recorded with a scanning rate of 1 nm by UV-Vis spectrophotometer (Shimadzu UV-2700). Fourier transform infrared (FTIR) spectra were collected on a Thermo Nicolet Nexus 470 FTIR. Photoluminescence (PL) spectra of the ZnO NPs dispersed in solvents were measured at 25 °C using a spectrofluorimeter (PerkinElmer LS 55, Xe flash lamp). The excitation and emission spectra were recorded in the range 200–800 nm under optimal conditions at maximum wavelengths for each studied system. The spectral data were acquired using 0.5-nm step and 3-nm bandwidths for both emission and excitation monochromators. X-ray diffraction (XRD) patterns were registered with CuKα radiation on a Phillips PW 1800 diffractometer.

The NLO response of solid precipitates, obtained from *i*-PrOH, EtOH, CH_3_CN, and HD, were studied within the self-action of picosecond laser pulses (42 ps FWHM, repetition rate 15 Hz) at 1064 nm [20, 21]. The precipitates were placed into the bed with ∅ ~ 8 mm in the Teflon® film positioned between two glass microscope slides (Marienfeld, 76 × 26 × 1 mm^3^). The film thickness *d* = 340 μm was chosen to provide an optical homogeneity of the solid sample layer sufficient for the registration transmittance level. This approach is described in detail for the carbon material bulk particle NLO diagnostics [22].

The obtained total and on-axis transmittance dependencies on the peak intensity *I* are presented as the smoothed curves produced by local B-splines of the measured data. Each curve corresponds to ~5000 registered laser shots. The relative errors of the curves do not exceed 0.2% for the total and 1% for the on-axis transmittance dependencies correspondingly.

**Fig. 1:**
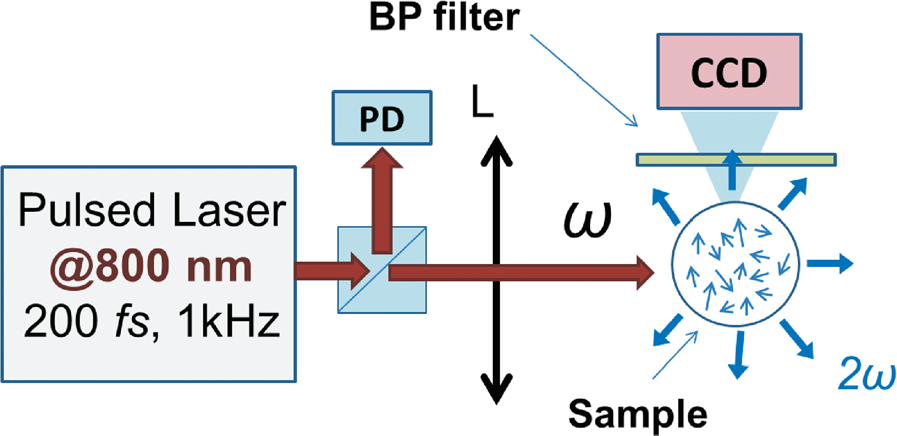
The experimental setup for the SHG efficiency measurements. PD - reference photodiode, BS - beam splitter, L - lens, CCD - CCD camera, BP - band-pass filter

The measurements of the SHG efficiency in the ZnO NPs solutions were performed within the excitation of femtosecond laser pulses (200 fs FWHM, repetition rate 1 kHz) at 800 nm. Figure 1 presents the scheme of the experimental setup.

The sample was positioned at the distance of 14.5 cm from the focusing lens L with a focal distance of 11 cm. The scattered second harmonic (SH) signal readout was realized at 90° from the laser beam axis by Atik 16IC CCD camera. In order to cut the fundamental radiation and extract the SH signal, band-pass (BP, 405 nm, 10-nm FWHM) filter was positioned on the CCD camera. The energy of the incident pump pulses was measured by the photodiode PD.

Taking into account that the displacement energy for ZnO is higher than 55 eV [23], we do not expect the formation of new vacancies and interstitials under the applied regimes of laser excitation in the experiments.

## Results and Discussions

**Fig. 2:**
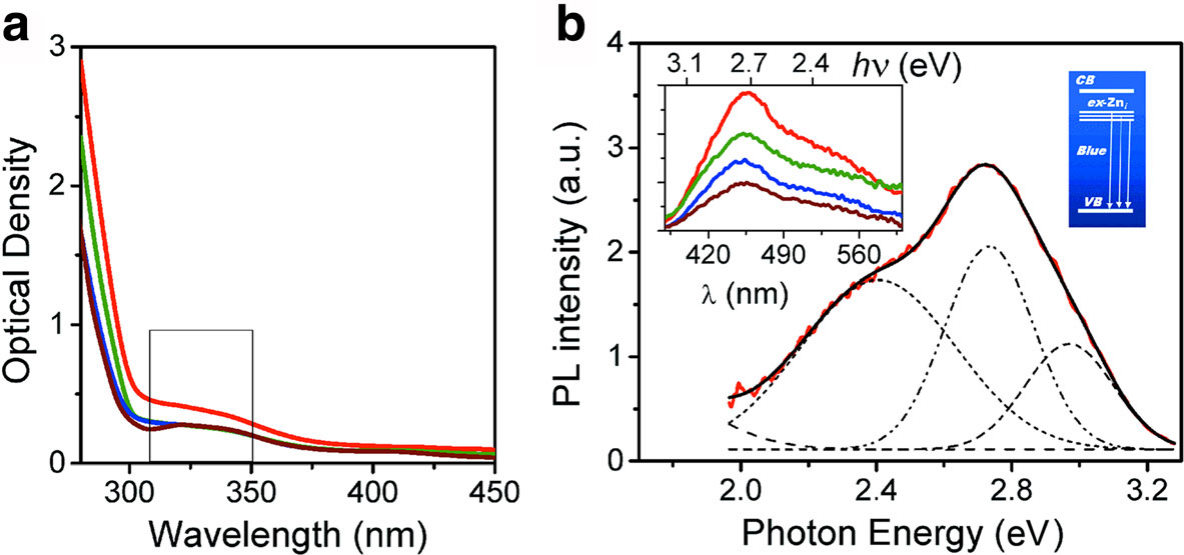
**a** UV-Vis of ZnO NPs in different solvents: EtOH (*red line*), i-PrOH (*green line*), MeCN (*blue line*), PPG (*brown line*). **b** PL spectrum of ZnO NPs in EtOH with Gaussian deconvolution (*dashed curves*); the *left insert* demonstrates the PL spectra of ZnO NPs in the solvents; the *right insert* shows the scheme of the radiative transitions in *blue*-*violet* range, *λ* - wavelength

Consistent with previous studies [6, 7, 14, 19], we observed that the core of ZnO was stabilized with TEA in abs. solvents, *i*-PrOH, EtOH, PPG, and MeCN, at the early stages of ZnAc_2_ hydrolysis. The ZnO NPs growth is effected by the high concentration of ZnAc_2_ together with TEA. Protecting the ZnO NPs core with adsorbates on the background of the increasing solution viscosity is sufficient to prevent the NPs from coagulation. Figure 2a shows UV-Vis spectra of the resulted ZnO NPs in different solvents. Typically, ZnO NPs size reduction causes the overall UV absorbance decrease and the broadening of the exciton-related peak at 360–370 nm [24]. In the presented spectra, we observe the smoothed adsorption peaks that provide reduced uniform absorption over the UV range and the blue spectral shift to ~330–350 nm. According to an approximation in [25], obtained ZnO NPs can be attributed to spherical particles with average diameter *D* of ~1.5–2.0-nm range. The impact of contrast viscosity of the resulted ZnO NP solutions could be a reason of the observed dispersion in the *D* values. Also, one should take into account that the absolute quantity of water is different in the used abs. solvents.

The ZnO NPs prepared were analyzed by excitation-emission spectrofluorimetry technique. PL spectra of ZnO NPs in different solvents show a broad emission ranging from 400 to 600 nm with the emphasis in the violet-blue range, Fig. 2b. The PL spectra were deconvoluted into Gaussian peaks at about 2.3, 2.8, and 3.0 eV (539, 443, and 413 nm, respectively). Several bands of the PL signal cannot be attributed to the presence of different NPs since the adsorption of acetate and TEA should prevent small NPs from aggregation. The PL intensity decreases, in the range of used solutions, from EtOH to PPG. This result correlates with the NPs concentration and UV-Vis absorption (cf. Fig. 2a, b). PL properties of ZnO, in general, are very sensitive to defects. The most probable explanation of observed PL spectra is the presence of numerous energy levels. Typically, the levels originated from various defects, including vacancies and interstitials.

According to Ref. [26], the green emission at ~2.3–2.4 eV (539–516 nm) is addressed to the transition from the conduction band (CB) to the deep-level states assigned to the oxygen vacancies (O_V_) of the NP interface. The violet and blue emission peaks were addressed to other defects [27–29]. The peaks at ~413 and 443 nm could be attributed to transitions between the highest inter-node and the lowest valence band (VB) [27]. Obviously, the intensive violet emission is due to defective zinc interstitial (Zn_*i*_) levels. The observed blue emission could be addressed to the different defects even in zinc vacancies as reported in [28].

The high intensity of the PL emission (see the left insert in Fig. 2b) indicates a high density of extended defects in the ZnO structure. This fact once again accentuates a non-equilibrium character of the NPs formation process.

**Fig. 3:**
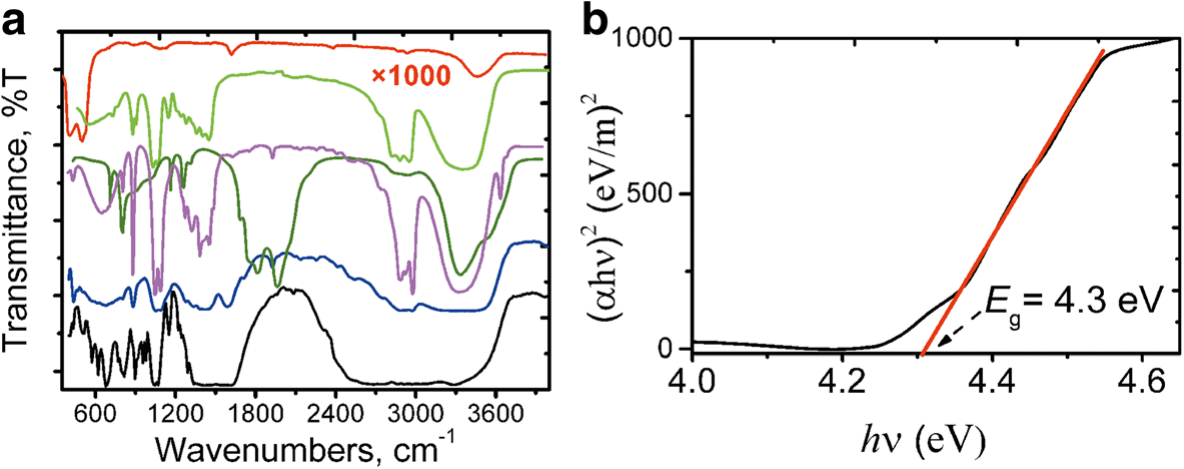
**a** FTIR spectra of ZnO NPs isolated from EtOH solution (*red line*); abs. EtOH (*pink line*); TEA (*yellow green line*); ZnO NPs in EtOH solution (*blue line*); all placed between KBr windows; ZnAc_2_ × 2H_2_O (*green line*) and ZnO NPs solid precipitate from EtOH (*black line*) (tablets in в KBr). **b** Tauc plot of (*αhν*)^2^ versus photon energy *hν* for residual ZnO NPs in abs. EtOH solution

We prepare three types of ZnO NPs-containing samples: ZnO NPs in the different solutions (before and after precipitation) and ZnO NPs dispersed in the precipitated solids. Figure 3a presents typical FTIR spectra of reagents, their mixture, and the reaction product, ZnO NPs solid isolated from EtOH. For spectra comparison needs, after the precipitate detachment, the ZnO NPs were centrifuged from the residual EtOH solution (at 10,000 rpm for 30 min), and separated sediment was washed thoroughly with abs. EtOH. The resulted yellow solid was dried in a hot air oven overnight at 60 °C.

For the isolated ZnO NPs, two efficient absorption bands were observed at about 407 and 503 cm^−1^. The band at 407 cm^−1^ was assigned to Zn–O stretching vibration, while the band at 503 cm^−1^ can correspond to the oxygen deficiency in ZnO NPs [30]. The H–O–H bending vibration mode is registered near 1620 cm^−1^. Asymmetric stretching vibrations of OH^−^ groups are identified in the range of 3210–3670 cm^−1^. These observations provided the presence of hydration in the structure. One could suppose the NPs capping agents such as Ac^−^ and TEA from the spectra comparison (Fig. 3a).

The UV-Vis spectra of solutions containing ZnO NPs and UV-DRS spectra of the co-precipitated mixture of the reagents and hydrolysis products (obtained from the same solutions) are similar (Additional file 1: Figure S3). A halo registered in the UV-DRS spectra is positioned in the same spectral range as bands in the UV-Vis spectra of ZnO NPs containing solutions that can be attributed to the ZnO NPs contribution.

After co-precipitation, the solutions were reexamined with UV-Vis spectroscopy (Additional file 1: Figure S4) and the recorded spectra were interpreted using a formula for the average particle radius [25]:

1 $$ r\;\left(\mathrm{nm}\right)=\frac{-0.3049+\sqrt{-26.23012+10240.72/\lambda}}{-6.3829+2483.2/\lambda}, $$

where *λ* corresponds to the peak position and indicates larger ZnO NPs in the range of *D* from 2.0 to 2.8 nm. This means coagulation of ZnO NPs with the smaller size (*D* ~1.5–2.0 nm) after the temperature reduction to 16 °C. A small area of the absorption peak and plenty of precipitated solid indicate that the remaining concentration of the ZnO NPs is very low. The NPs growth process does not reach the stoichiometric composition of NPs cores that was confirmed by the blue emission at about 443–450-nm manifestation in the PL spectra (Fig. 2b). At the background of the stabilization with the same amine, the size of ZnO NPs is weakly dependent on the used solvents.

The optical bandgap energy (*E*_g_) was estimated from Tauc plots (Fig. 3b, Additional file 1: Figure S3). To estimate the bandgap shift with the NPs size, one can use the cluster size equation from Jortner’s work [31]. In the case of ZnO NPs, we used the modified form of the equation proposed in [32]:

**Fig. 4:**
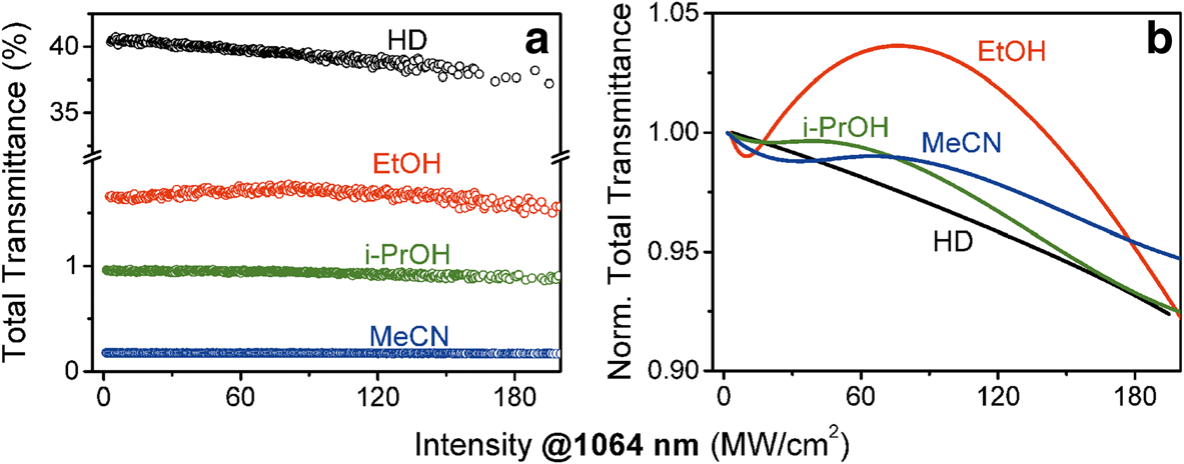
The photoinduced variations of the total transmittance due to the self-action of the picosecond laser pulses at 1064 nm for the ZnO NPs precipitates, obtained from different solvents. **a** The total transmittance versus the peak laser intensity. **b** The same smoothed total transmittance dependencies that were normalized on *T*_0_ (see Table 1) — the transmittance in a linear regime

2 $$ {E}_{\mathrm{g}} = 3.35 + 100\cdot {\left(18.1\cdot {D}^2+41.4\cdot D\hbox{--} 0.8\right)}^{-1} $$

In contrast to the Brus equation [33] that can be applied for larger NPs of above 6 nm in diameter, the range of validity of Eqn. (2) covers the NPs sizes from *D* ~2.5 to 6 nm [32]. However, in the present case, the value of *E*_g_ was found within a range of 4.10 to 4.72 eV that corresponds to the NPs sizes varying from *D* ~1.8 to 1.2 nm, which is far below the recommended interval.

Summing up the observations collected during the preparation of the solid precipitates, we will briefly discuss the findings below. Undoubtedly, the ZnO NPs have the potential to be immobilized by occlusion — the incorporation of nanoparticles inside the growing solid. Typically, the precipitate, at the initial formation stage, captures the small ZnO NPs, of ca. 2–3 nm, contained in the solutions. The fast rate of the process prevents further NPs growth. It resulted in the formation of the solids containing defective ZnO NPs, which are similar to the ones reported in [34, 35]. In contrast to the small ZnO NPs, the solids with HD contains 50 nm ZnO covered with HD and the rest of the hydrolysis products.

In comparison to good-quality XRD patterns reported in [32, 36], the XRD patterns obtained in this work are mainly of a lower quality. We assume this is due to the semi-amorphous origin of the solids and a low content of ZnO NPs covered with a mixture of reagents and products.

It should be noted that the NLO characterization of the ZnO NPs precipitates was done at the wavelength 1064 nm (1.17 eV). The laser quanta 1.17 eV can induce resonant two-photon absorption transitions into the V_O_ band with a peak at about ~2.4 eV. The precise peak position and the area obtained from the Gaussian decomposition of the PL spectra are presented in Table 1.

**Table 1:** PL and NLO parameters

Solvent	Parameters of V_O_ band		NLO parameters		
	Peak position, eV	Area, a.u.	*T*_0_, %	Re(*χ* ^(3)^), ×10^−9^ esu	Im(*χ* ^(3)^), ×10^−11^ esu
EtOH	2.41	0.93	1.7	2.4	1.4
*i*-PrOH	2.44	0.56	1.0	−4.2	0.7
MeCN	2.34	0.28	0.2	2.9	0.5
HD	–	–	40.5	−7.1	0.4

The transmittance in a linear regime *T*_0_, the effect of the solution on the V_O_ band (~514–530 nm) peak position and area obtained from the Gaussian decomposition of the PL spectra, the real and imaginary parts of the cubic NLO susceptibility *χ* ^(3)^ for the ZnO NPs precipitates obtained from different solvents

Figure 4a presents the photoinduced variations of the total transmittance of the solid precipitates, obtained from *i-*PrOH, EtOH, MeCN, and HD. It was shown the total transmittance variation in the studied peak intensity range to be slow. At the initial *I* ≤ 2 MW/cm^2^ range, the total transmittance was approx. constant (*T*_0_) that corresponded to the linear regime of the optical transmittance. The *T*_0_ magnitude significantly depends on the solvent; see Table 1. In the range of used solvents, the magnitude reduces in the following order HD > EtOH > *i*-PrOH > MeCN. To derive the photoinduced effect manifestation from the linear response of a background, we normalized the obtained dependencies (Fig. 4a) on a linear transmittance *T*_0_. The results are shown in Fig. 4b as smoothed curves with the relative error of about ±0.2% [22].

Analysis of the presented data reveals non-monotonic photoinduced total transmittance variations in precipitates from EtOH, *i*-PrOH, and MeCN, while for the case of HD, monotonic photoinduced darkening was observed. For the peak intensity range >100 MW/cm^2^, the efficient photodarkening for all studied samples can be attributed to the two-photon resonant excitation of the V_O_. For this range, from the obtained dependencies, the imaginary part of the cubic NLO susceptibility Im(*χ* ^(3)^) was estimated within the approach described in [20]. The results are presented in Table 1. One can see the Im(*χ* ^(3)^) magnitudes decrease in the following order EtOH > *i*-PrOH > MeCN that correlates with the V_O_ band area, being proportional to the NPs concentration.

The on-axis transmittance dependencies reveal the different sign of the refractive NLO response for the ZnO NPs precipitates, obtained from different solvents. The photoinduced self-focusing effect was observed for MeCN and EtOH while the self-defocusing one for HD and *i*-PrOH. From the obtained dependencies, the real part of the cubic NLO susceptibility Re(*χ* ^(3)^) was estimated [22]. The observed Re(*χ* ^(3)^) positive magnitudes for the ZnO NPs precipitates, obtained from MeCN and EtOH, can be explained by the two-photon resonant excitation of the oxygen vacancies. The two quanta energy 2.33 eV does not exceed the V_O_ band spectral position (see Table 1) that determines the positive refractive NLO response of the trapped carriers [37]. In this case, a more efficient refractive response of ZnO NPs in MeCN is dealing with better overlapping of the V_O_ band position with the two-photon laser quanta energy.

The manifestation of the self-defocusing effect (Re(*χ* ^(3)^) < 0) for the ZnO NP precipitate obtained from *i*-PrOH solutions requires more detailed study.

**Fig. 5:**
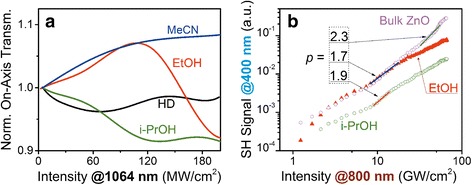
**a** The photoinduced variations of the on-axis transmittance due to the self-action of picosecond laser pulses at 1064 nm for the ZnO NPs precipitates, obtained from different solvents. **b** The SHG signal in ZnO NPs solutions and colloidal suspension (commercial bulk ZnO) versus the peak intensity of the pump femtosecond laser pulses at 800 nm

We have also performed the SHG efficiency measurements for ZnO NPs in EtOH and *i*-PrOH within femtosecond laser pulse excitation at 800 nm. The results were compared with the colloidal suspension of large ZnO NPs in EtOH, to be referred to as bulk ZnO. This suspension was prepared from the NPs with different sizes ranging from 90 to 200 nm, as in [17]. The average particle size was ~150 nm. In such NPs, the SHG efficiency is determined by the electric dipole mechanism and proportional to the square volume. The SH signal dependencies are presented in Fig. 5b in a log–log scale.

In order to compare the results, we analyzed the SHG efficiency in certain excitation ranges. The effective order *p* of the NLO response *I*_2ω_ ~ (*I*_ω_)^*p*^ was estimated from the slope of the linear approximation of the presented dependencies. At peak intensity range 10–13 GW/cm^2^, the maximal *p* = 1.9 was obtained for the *i*-PrOH solution containing ZnO NPs. The synthesized and large ZnO NPs demonstrated lower *p* = 1.7 in the EtOH. Higher SHG efficiency in the small ZnO NPs instead of the large ones indicates the manifestation of the surface mechanisms on the background of the bulk response.

For *I*_ω_ > 15 GW/cm^2^, we have observed higher SH magnitude from the larger NPs with *p* = 2.3. It can be explained by the cubic NLO response contribution into the SHG efficiency enhancement. The similar effect was observed in the KDP single crystals with incorporated TiO_2_ NPs [38].

The obtained result indicates the high brightness of the SHG signal of the synthetized novel small (~2 nm) ZnO NPs, making them promising for biolabeling application [39].

## Conclusions

A set of the ZnO NPs with efficient luminescent and quadratic NLO responses was successfully prepared by the hydrolytic route. The ZnO NPs demonstrated the emission in the solution and in the solid state. The solutions and solid precipitates containing ZnO NPs were characterized with UV-Vis/UV-DRS and FTIR spectroscopy. Small ZnO NPs were stabilized at the early stages of hydrolysis by protecting NPs cores with adsorbates and limiting the particles’ growth. The analysis of UV-Vis absorption in the range of 330–350 nm revealed the obtained ZnO NPs sizes within 2.0–2.8 nm.

The PL emission and UV-Vis absorption manifestation with increases symbate with the concentration of ZnO NPs. It grows in the following order of the solvents EtOH > *i*-PrOH > MeCN > PPG. The violet, blue, and green PL band emissions from the solutions were addressed to the NPs with high-defect concentration.

The NLO characterization of the ZnO NPs containing precipitates was provided by the self-action effect monitoring within the picosecond laser pulses at 1064 nm. It was shown that the ZnO NPs containing precipitates obtained from different solvents produce a different efficiency of the absorptive and refractive NLO responses. The correlation between the Im(*χ* ^(3)^) magnitudes and the area of the PL band at about 2.34–2.41 eV that related to the oxygen vacancies in ZnO was revealed. These results indicate high potential of the applied technique for the ZnO NPs diagnostics.

The second harmonic generation in the ZnO NPs was studied within the femtosecond laser excitation at 800 nm. It was shown that the SHG efficiency of the synthetized ZnO NPs (~2 nm) and the large commercial ZnO NPs (~150 nm) are comparable. The effect is important for the application of small ZnO NPs in biolabeling.

## Additional file

Additional file 1: Figure S1. A typical color change of EtOH (a–c) and (d–e) *i*-PrOH solutions of Zn(CH_3_COO)_2_ × 2H_2_O, at different stages of the interaction of components: (a) starting solution 1–2 min after the preparation, (b) 30 min, and (c) 1-h heating at 60 °C; (d) 20 min after the preparation, (e) 60 min, and (f) 2-h heating at 60 °C. **Figure S2.** The starting *i*-PrOH solution of Zn(CH_3_COO)_2_ × 2H_2_O (a) and (b–c) luminescence of ZnO NP obtained by hydrolysis. **Figure S3** a, b. Comparison of UV-Vis spectra of ZnO NP in solutions and solid precipitates. **Figure S4.** UV-Vis spectra of ZnO NP in the resulted solutions (a) EtOH, (b) *i*-PrOH, (c) MeCN, and (d) PPG. (PDF 346 kb)
