# Supplementary material for: Synthesis, Characterization, Luminescent and Nonlinear Optical Responses of Nanosized ZnO
Source: Nanoscale Res Lett. 2017 Mar 3;12:164. doi: 10.1186/s11671-017-1934-y (PMC5334190; doi:10.1186/s11671-017-1934-y)
Supplement: Additional file 1: Figure S1. — A typical color change of EtOH (a–c) and (d–e) i-PrOH solutions of Zn(CH3COO)2 × 2H2O, at different stages of the interaction of components: (a) starting solution 1–2 min after the preparation, (b) 30 min, and (c) 1-h heating at 60 °C; (d) 20 min after the preparation, (e) 60 min, and (f) 2-h heating at 60 °C. Figure S2. The starting i-PrOH solution of Zn(CH3COO)2 × 2H2O (a) and (b–c) luminescence of ZnO NP obtained by hydrolysis. Figure S3 a, b. Comparison of UV-Vis spectra of ZnO NP in solutions and solid precipitates. Figure S4. UV-Vis spectra of ZnO NP in the resulted solutions (a) EtOH, (b) i-PrOH, (c) MeCN, and (d) PPG. (PDF 346 kb) [file 11671_2017_1934_MOESM1_ESM.pdf]

Electronic Supplementary Material

Synthesis, characterization, luminescent and nonlinear optical responses of nanosized ZnO

Volodymyr V. Multian[1], Andrii V Uklein[1], Alexander N. Zaderko[2], Vadim O. Kozhanov[2], Olga Yu. Boldyrieva[2], Rostyslav P. Linnik[2], Vladyslav V. Lisnyak[2], Volodymyr Ya. Gayvoronsky[1]

[1]Institute of Physics, the National Academy of Science of Ukraine, pr. Nauky, 46, 03028 Kyiv, Ukraine.
[2]Chemical Faculty, Taras Shevchenko National University of Kyiv, 62a, Volodymyrska Str., 01601 Kyiv, Ukraine

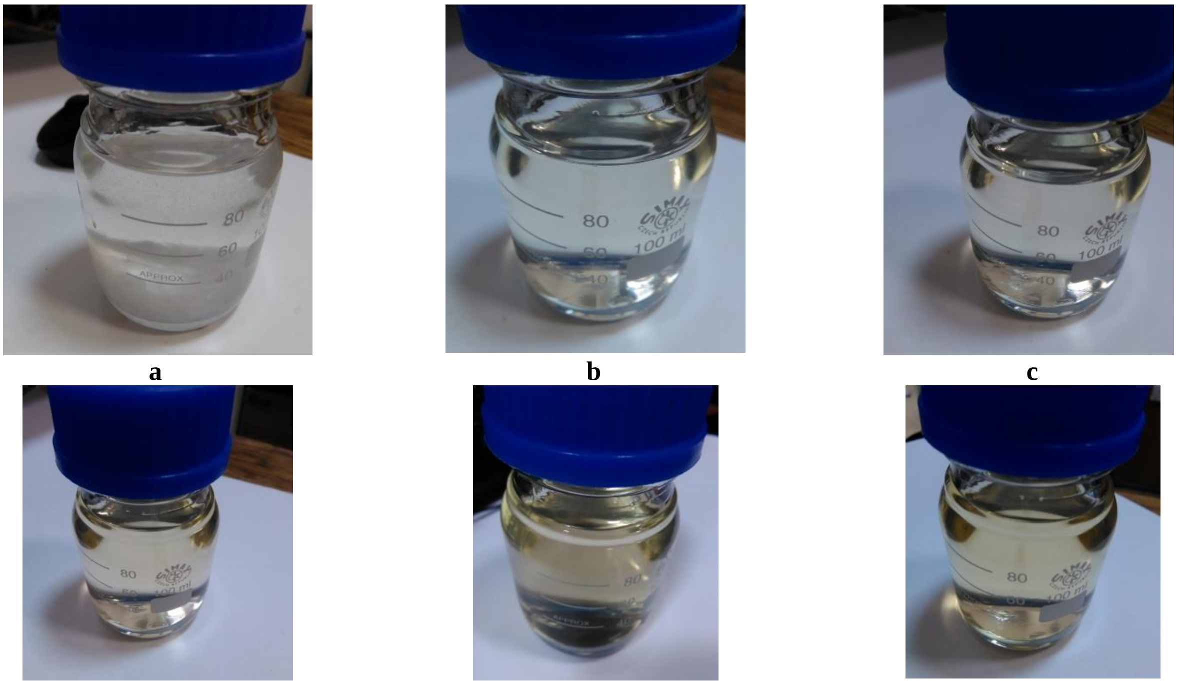

a b c

d e f

Figure S1 A typical color change of EtOH **a-c** and **d-e** i-PrOH solutions of $Zn(CH_3COO)_2 \times 2H_2O$, at different stages of the interaction of components: **a** starting solution 1–2 min after the preparation, **b** 30 min and **c** 1 h heating at 60 °C; **d** 20 min after the preparation, **e** 60 min and **f** 2 h heating at 60 °C

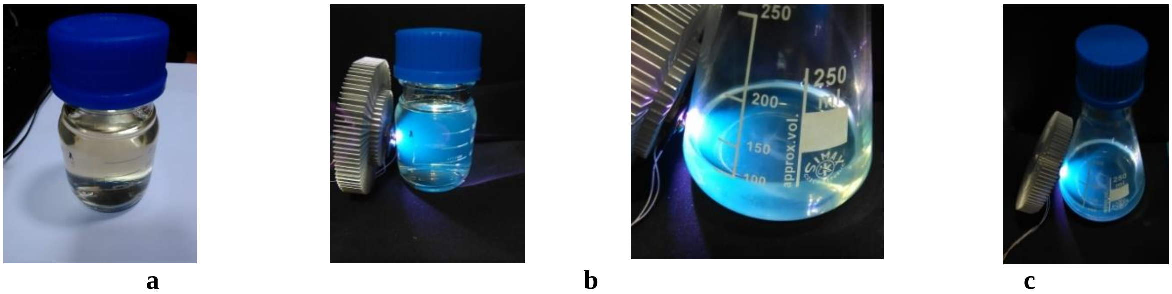

a b c

Figure S2 The starting i-PrOH solution of $Zn(CH_3COO)_2 \times 2H_2O$ **a** and **b-c** luminescence of ZnO NP that obtained by hydrolysis

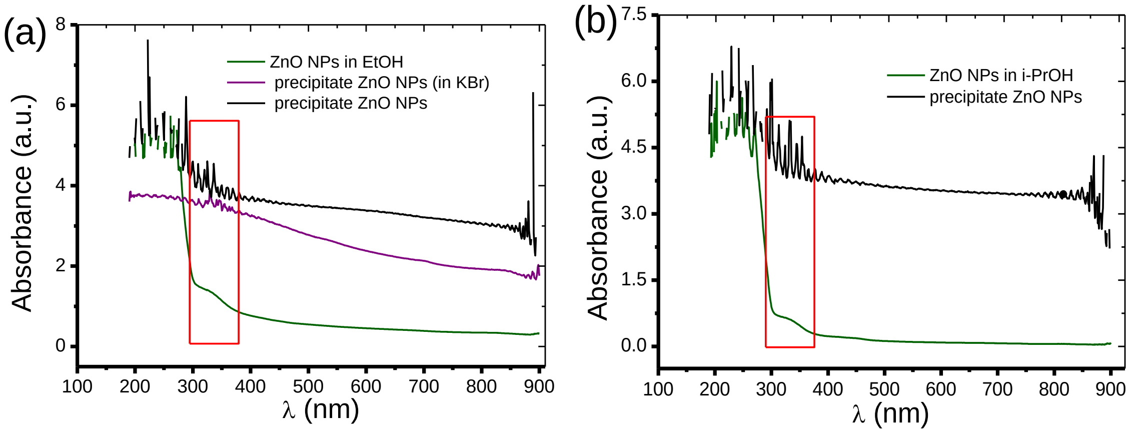

Figure S3 a, **b** Comparison of UV-Vis spectra of ZnO NP in solutions and solid precipitates

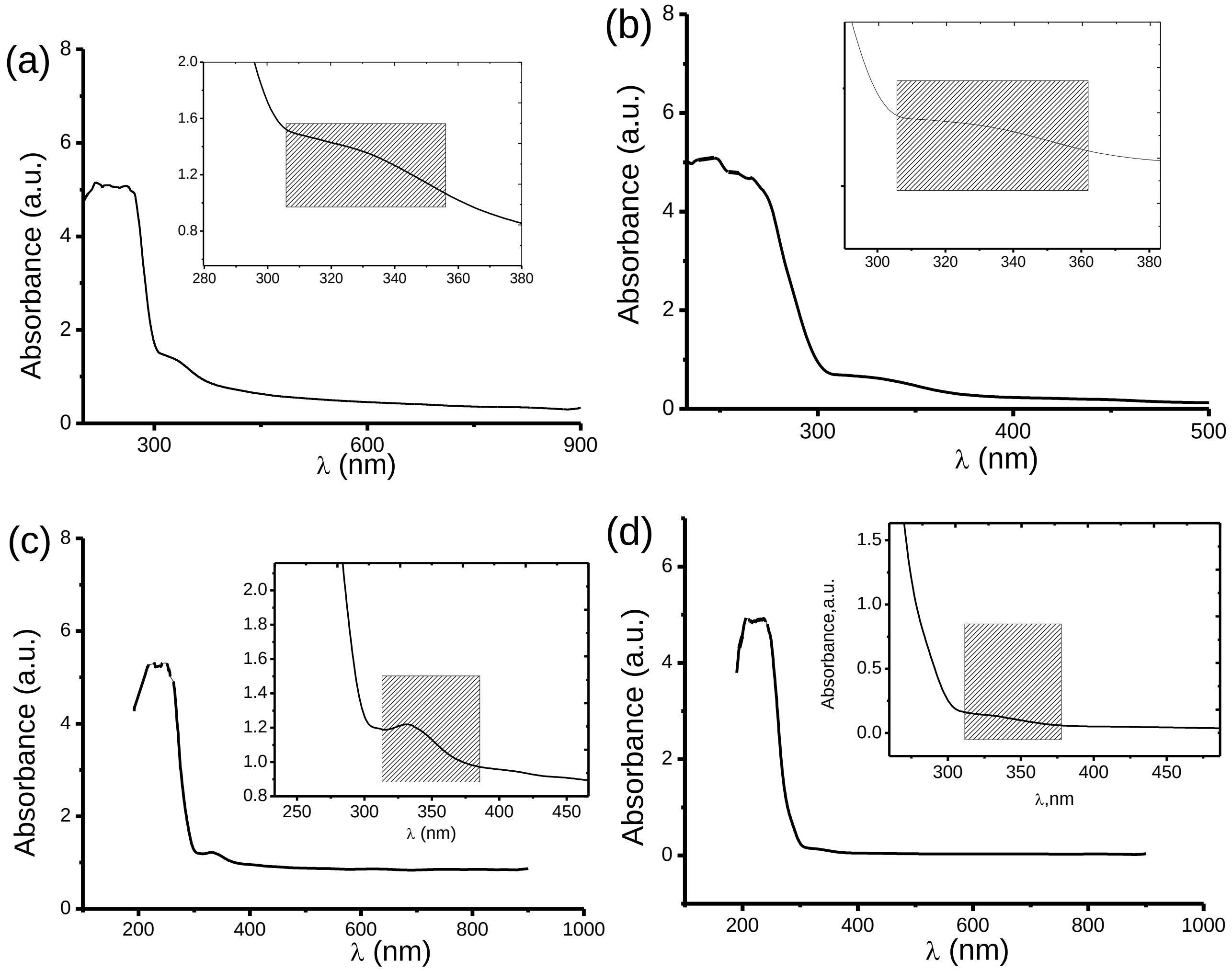

Figure S4 UV-Vis spectra of ZnO NP in the resulted solutions **a** EtOH, **b** i-PrOH, **c** MeCN, **d** PPG
